# 542 Identifying Psychosocial Factors Impacting Burn Survivors

**DOI:** 10.1093/jbcr/irad045.139

**Published:** 2023-05-15

**Authors:** Debra Kilmer, Paula Cottrill, Amanda Rose

**Affiliations:** Akron Children's Hospital, Akron, Ohio; Akron Children's Hospital, Akron, Ohio; Cleveland Clinic Akron General & Akron Children's Hospital, Akron, Ohio; Akron Children's Hospital, Akron, Ohio; Akron Children's Hospital, Akron, Ohio; Cleveland Clinic Akron General & Akron Children's Hospital, Akron, Ohio; Akron Children's Hospital, Akron, Ohio; Akron Children's Hospital, Akron, Ohio; Cleveland Clinic Akron General & Akron Children's Hospital, Akron, Ohio

## Abstract

**Introduction:**

Burn survivors often suffer from profound psychosocial stress. Following a traumatic burn, new stressors and mental health symptoms can arise, and previously existing factors can be amplified. Alleviation of these stressors can facilitate healing, treatment compliance, and favorable outcomes.

We sought to better identify psychosocial factors that impact the lives of adult burn survivors. Our primary aim was to develop a psychosocial screening battery and brief screener to identify stressors common among such patients at our hospital-based outpatient burn center.

**Methods:**

A survey was administered to adult burn survivors at a monthly support group. Participants were instructed to endorse items from a list of 39 possible stressors and symptoms that impacted them after their burn injury. The most frequently endorsed items were considered for inclusion in the screening tool.

**Results:**

A total of 17 surveys were completed, nine (53%) of whom were men and eight (47%) of whom were women. Ages ranged from 18 to 79 years. The earliest burn injury occurred in 1990, and the most recent occurred in 2022. There were 17 survey items endorsed by 29% or more of the respondents (Table 1).

**Conclusions:**

Numerous symptoms and stressors endorsed by participants align with mental health conditions like acute stress disorder, post-traumatic stress disorder (PTSD), anxiety, and depression. We decided to implement the Primary Care PTSD Screen and the Generalized Anxiety Disorder 7-Item within our outpatient clinic in addition to the depression screen already in use. Items not addressed by questions on these mental health screens were included in our brief burn survivor stress screening measure.

**Applicability of Research to Practice:**

Implementation of this screening battery will enable identification of psychosocial factors impacting adult burn patients previously hospitalized as a result of their injuries. This will allow us to address patient concerns, offer recommendations for support and resources, and guide treatment planning to improve compliance and outcomes. In the future, this information will facilitate the development of additional programs to assist burn survivors.

